# TAAR9 knockout increases hippocampal serotonin and alters grooming behavior in rats

**DOI:** 10.3389/fphar.2025.1684029

**Published:** 2025-11-12

**Authors:** Ilya S. Zhukov, Inessa V. Karpova, Ramilya Z. Murtazina, Yazen Alnefeesi, Olga M. Korenkova, Ilia Yu. Tissen, Svetlana A. Palchikova, Lydia A. Tokareva, Sarng S. Pyurveev, Petr D. Shabanov, Larisa G. Kubarskaya, Mikhail A. Rozhko, Ekaterina B. Zernova, Ekaterina A. Zolotoverkhaja, Anna B. Volnova, Allan V. Kalueff, Natalia V. Alenina, Raul R. Gainetdinov

**Affiliations:** 1 Institute of Translational Biomedicine, Saint Petersburg State University, Universitetskaya nab., Saint Petersburg, Russia; 2 Institute of Experimental Medicine, Saint Petersburg, Russia; 3 Institute of Toxicology of Federal Medical-Biological Agency, Saint Petersburg, Russia; 4 Suzhou Key Laboratory of Neurobiology and Cell Signaling, Department of Biosciences and Bioinformatics, School of Science, Xi’an Jiaotong-Liverpool University, Suzhou, China; 5 Max Delbrück Center for Molecular Medicine in the Helmholtz Association (MDC), Berlin, Germany; 6 Saint Petersburg State University Hospital, Saint Petersburg State University, Universitetskaya nab., Saint Petersburg, Russia

**Keywords:** trace amines, trace amine-associated receptor, TAAR9, expression, behavior, neurochemistry, rat knockout model, GPCR

## Abstract

**Introduction:**

Despite their association with brain disorders, the neurophysiological roles of the trace amine-associated receptors remain poorly understood. In humans, the genomic *trace amine-associated receptor* cluster comprises nine consecutive genes, six of which code for functional proteins (TAAR1, TAAR2, TAAR5, TAAR6, TAAR8, TAAR9). While homologues of the former three are known to regulate classical monoamines and neurogenesis, the functions of the latter three remain largely unknown. In this exploratory study, we demonstrate for the first time that TAAR9 plays a significant regulatory role in the monoaminergic systems of the rat.

**Methods:**

We used qPCR to measure TAAR9 mRNA expression throughout the rat brain. Serotonin, dopamine, and their metabolite levels were assessed by HPLC in brain tissues from TAAR9-KO and wild-type littermates. We applied fast-scan cyclic voltammetry to measure mesolimbic dopamine release. Behavioral analysis included assessment of grooming, anxiety-like, and sexual behaviors. A battery of hematological/hormone assays was also applied.

**Results and discussion:**

We detected TAAR9 mRNA in the brainstem and midbrain–regions that include key monoaminergic nuclei such as the locus coeruleus, raphe nuclei, and the ventral tegmental area. The TAAR9-KO rats exhibited increased hippocampal serotonin levels and a slight shift in dopamine turnover, but not mesolimbic dopamine release. Although hippocampal serotonin is commonly implicated in mood and anxiety regulation, behaviorally, no genotype differences were detected in the elevated plus maze, suggesting that basal anxiety-like behavior remained unaffected under the test conditions. However, changes in grooming microstructure indicated subtle alterations in behavioral organization, which may reflect the neurochemical changes observed in the hippocampus. No changes were evident in a battery of hematological assays.

**Conclusion:**

Together, these findings suggest that TAAR9 deletion selectively modulates central monoaminergic systems and related behavioral patterns, without altering systemic physiological parameters.

## 1 Introduction

Despite long-established links to human brain disorders (Berry et al., 2017; Boulton, 1974; Grandy, 2007), the physiology of the trace amines and their receptors remains largely uncharacterized (Alnefeesi et al., 2021; Berry et al., 2017). Endogenously produced *via* amino acid decarboxylation (Gainetdinov et al., 2018; Premont et al., 2001), trace amines include β-phenylethylamine, p-tyramine, tryptamine, and p-octopamine (Gainetdinov et al., 2018). These compounds arise in the metabolic pathways of the monoaminergic neurotransmitters, yet their brain concentrations are approximately 100-fold lower (Berry, 2004; Boulton, 1974). Some members of the trace amine-associated receptor (TAAR) family have been shown to modulate the classical monoamine systems, potentially by heterodimerization with other aminergic receptors (Borowsky et al., 2001; Bunzow et al., 2001). In humans, the genomic TAAR locus includes nine consecutive genes of which six code for functional proteins (TAAR1, TAAR2, TAAR5, TAAR6, TAAR8, TAAR9), and three represent pseudogenes (Eyun et al., 2016). Transcripts of the first four functional TAARs have been detected in the limbic system (Katolikova et al., 2022), and all six are widely expressed in the periphery (Revel et al., 2013; Rutigliano et al., 2018). Despite a paucity of research on TAARs two to nine, and the typically low and inconsistent mRNA levels in the brain, multiple studies have reported significant–yet poorly understood–neurological effects. Earlier studies identified the TAARs as chemosensory G-protein coupled receptors (GPCRs) within the olfactory epithelium, supporting the then-prevailing notion that their neural functions were limited to olfaction (Li et al., 2012; Li et al., 2015; Liberles and Buck, 2006).

Thus far, the most studied member of the TAAR family has been TAAR1 (Alnefeesi et al., 2021), which is uniquely absent in olfactory epithelia (Liberles and Buck, 2006). The TAAR1 agonist ulotaront has yielded positive results in both preclinical and clinical studies (Kuvarzin et al., 2023), which show a favorable ratio of antipsychotic efficacy to adverse events (Le et al., 2023). Since the first of the clinical studies, the psychiatric potential of TAAR1 has gradually expanded beyond the treatment of psychosis, with prospects in the treatment of mood and anxiety disorders (Alnefeesi et al., 2021; Alnefeesi et al., 2024). More recently, independent preclinical studies have shown that TAAR1 agonism can abolish two distinct forms of abnormal aggression (Wang et al., 2024; Zhukov et al., 2024), suggesting utility in antisocial, bipolar, and personality disorders. Knockout of TAAR1 in mice elicits significant changes in grooming microstructure and increases aggressive behaviors (Zhukov et al., 2022a). In line with this, the TAAR1 agonist RO5263397 reduces aggression in brain serotonin-deficient tryptophan hydroxylase 2 knockout rats (Zhukov et al., 2024). Independent work also found potential utility for TAAR1 as a novel pharmacological target for multiple sclerosis and neuroinflammation (Barnes et al., 2021; Barnes et al., 2023). These results on TAAR1 have been adequately explained by well-established neuromodulatory and neurotrophic effects in monoaminergic regions such as the ventral tegmental area (VTA) and the dorsal raphe nuclei (DRN) (Revel et al., 2013; Shi et al., 2019).

Further progress has increasingly revealed that features once thought to be unique to TAAR1 are shared by other TAARs as well. Transcripts for TAARs 1, 2, 5, and 6 have now been detected in several limbic brain regions known to play critical roles beyond olfaction (Katolikova et al., 2022; Li and Liberles, 2016). Although transcript detection is rare in most datasets, the ample links to psychiatric disorders suggest low mRNA-protein correlations, possibly due to oscillatory or burst-like transcriptional dynamics (see Koussounadis et al., 2015; Lee et al., 2023 for context). Such periodic transcription is well documented for genes that regulate proliferation and differentiation processes including those related to cancer and neurogenesis (Dominguez et al., 2016; Kageyama et al., 2020). Knockout of TAAR2 in mice increases the number of dopaminergic neurons in the substantia nigra, in addition to neuroblast-like and proliferating cells in the subventricular (SVZ) and subgranular (SGZ) zones (Efimova et al., 2022). Similar results are evident in TAAR5-KO mice (Efimova et al., 2021). Mouse TAAR2 and TAAR5 are enriched not only in olfactory areas but also in deeper regions of the limbic system, again suggesting neurological roles beyond olfaction (Espinoza et al., 2020). More recently, TAAR1 was also shown to regulate neurogenesis in the SGZ (Zhang et al., 2024), and other studies implicate the TAARs in tumor growth (Park et al., 2024; Pitts et al., 2019; Vaganova et al., 2022; Vaganova et al., 2023; Vogelsang et al., 2021). The family-wise pattern thus shows a clear involvement in growth processes which lend themselves to periodic transcription. This would explain the co-occurrence of robust neurological effects with inconsistent/low mRNA levels.

**FIGURE 1:**
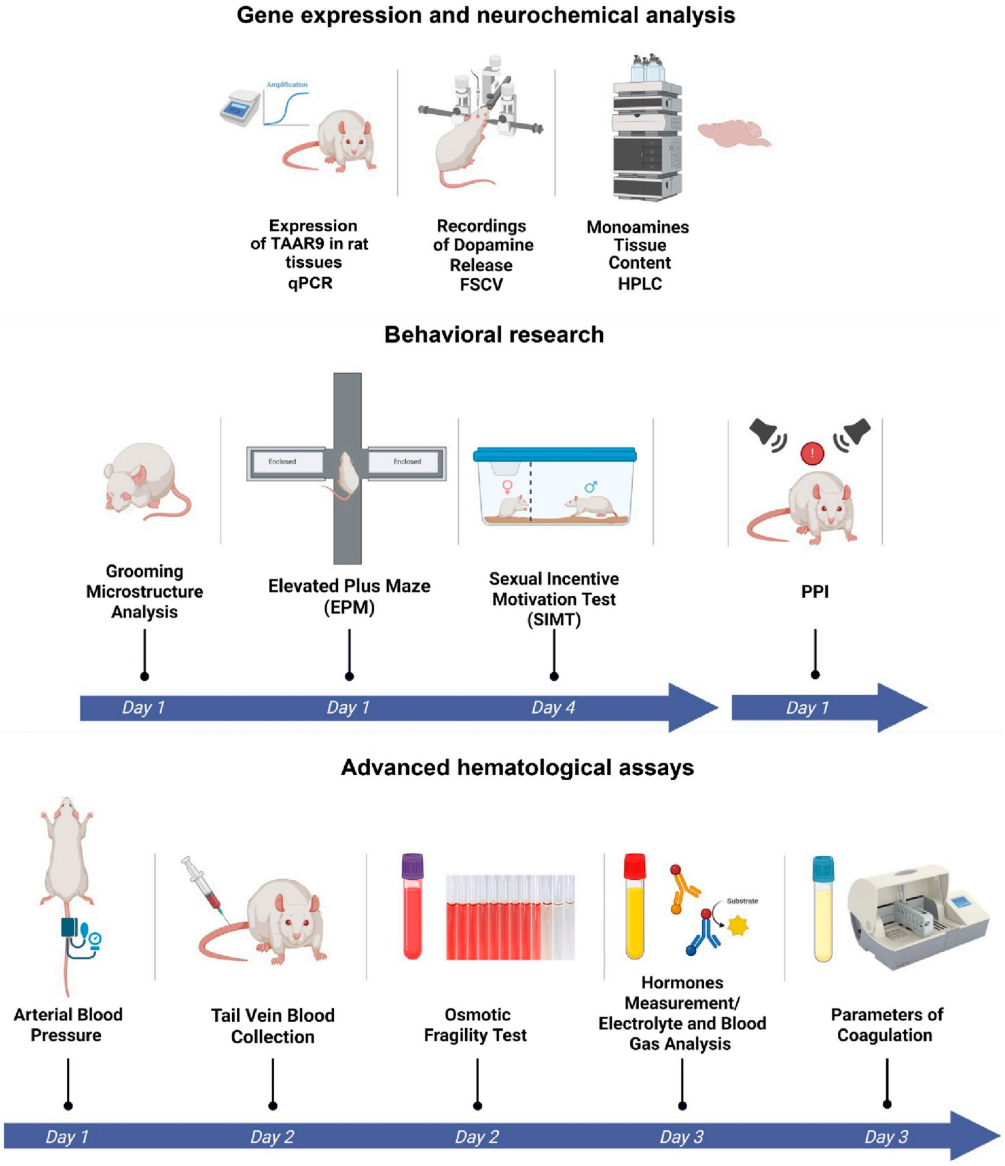
Experimental design of comparative analysis between WT and TAAR9-KO rats. Broad characterization included: evaluation of *Taar9* expression in Sprague-Dawley rats, neurochemical and behavioral experiments and advanced hematological assays.

In this study, we continue to characterize the TAAR9-KO rat line (Murtazina et al., 2021) to further define the physiological functions of TAAR9. We had previously identified significant peripheral changes in these rats, including reduced blood LDL cholesterol levels (Murtazina et al., 2021) and a shift in the gut microbiota (Zhukov et al., 2022c). The present study included three sets of exploratory assays that compared the TAAR9-KO rats with wild-type (WT) counterparts. The first set quantified the relative expression of TAAR9 transcripts in the brain and adrenal gland, the second explored changes in brain monoamine levels and behavior, and the third involved broad-sweeping hematological assays. The full experimental design is outlined in Figure 1.

## 2 Materials and methods

### 2.1 Animals

The TAAR9-KO^delC^ strain (TAAR9-KO hereafter) was originally generated using CRISPR/Cas9 and backcrossed to the outbred Sprague-Dawley (SD) genetic background over 6 generations. All TAAR9-KO rats reported on herein are of the TAAR9-KO^delC^ strain (Murtazina et al., 2021). Adult wild-type (WT) and TAAR9-KO male rats (35 weeks old) were born from heterozygous parents and genotyped according to the previously described protocol (Murtazina et al., 2021). Rats were maintained under standard lab conditions (room temperature and humidity 21 °C ± 5 °C and 40%–70%, respectively) on a 12:12 h light/dark cycle with a light on at 10:00 h and allowed access to food and water *ad libitum*. All experiments were conducted during the light phase from 15:00 to 22:00. The rats were acclimated to the experimental room for at least 1 h prior to behavioral testing. All rats were genotyped before and after the experiments (i.e., post mortem). All procedures were performed under the guidelines established by the European Community Council (Directive 2010/63/EU of 22 September 2010) and animal protocols were approved by the responsible governmental authorities (Landesamt für Gesundheit und Soziales (LaGeSo), Berlin, Germany, and Ethics Committee of St. Petersburg State University, St. Petersburg, Russia).

### 2.2 Group independence and overlap

The total number of adult male rats used in the present study was N = 82; these were all of the same age. A total of 3 WT rats were used to quantify TAAR9 expression. A total of 13 rats were used in behavioral assays: TAAR9-KO (n = 7) and WT (n = 6). After a minimum 5 days, and a maximum of 9 days since the last behavioral assay, these animals were assessed by *in vivo* FSCV. Two animals from each group died in the process, which left a total of eight rats for FSCV assays (n = 4 per genotype). To prevent potential stress from behavioral studies, an independent cohort of 22 rats was used for HPLC measurements of monoamine tissue content: TAAR9-KO (n = 11) and WT (n = 11). Assessment of prepulse inhibition (PPI) was carried out on another independent cohort of 23 rats in total: TAAR9-KO (n = 11) and WT (n = 12). For hematological tests we pooled results from yet another pair of groups (Total N = 24 rats).

### 2.3 *Taar9* expression in rat tissues

The expression of TAAR9 in rats’ different brain regions and adrenal glands of SD-Hannover rats (JANVIER LABS) was assessed using q-PCR (n = 3 per group). After removal, the tissues were snap-frozen on dry ice and stored at −80 °C. Total RNA was isolated using the Trizol reagent according to the protocol of the manufacturer. RNA samples were treated with DNase I (Sigma-Aldrich) to remove residual genomic DNA. cDNA was produced from 1 μg of DNaseI-treated RNA using Moloney Murine Leukemia Virus (M-MLV) reverse transcriptase (Promega Corporation). Quantitative PCR was performed with SYBR green master mix reagents (Thermo Fisher Scientific) using the QuantStudio™ 5 Real-Time PCR System (Thermo Fisher Scientific) with TAAR9_specific primers (fw: 5′- GGA ACT TAC TGG TCA TCA CCG C and rev: 5′- CTA AAG GGC ATC ACA GTC ACC C) and normalized to the expression of the housekeeping gene TATA-Box binding protein (TBP) using the 2^−ΔΔCT^-method.

### 2.4 Neurochemical assays

HPLC Measurements of the Monoamines Tissue Content was performed to detect a group of monoamines and their metabolites: NE, DA, 5-HT, 3,4-dihydroxyphenylacetic acid (DOPAC), homovanillic acid (HVA) and 5-hydroxyindoleacetic acid (5-HIAA). Detection was performed on a Shimadzu LC-20 Prominence chromatograph (Shimadzu, Japan) with a Decade Elite electrochemical detector (Antec, Netherlands). The chromatographic system included a Rheodyne 7125 injector (Rheodyne LLC, United States) with a 20 µL loop for the sample application and a Phenomenex column (4.6 × 150.0 mm) with a Sphere Clone 5 u ODS (2) sorbent (Phenomenex Inc., United States). Different brain structures (olfactory tubercle, hippocampus, striatum, and cerebral cortex) were isolated from the right hemisphere, prepared on ice, frozen in liquid nitrogen, and stored at −80. The homogenization of the samples was carried out in liquid nitrogen at −198 °C with CryoMill (Retsch, Germany). The samples were then dissolved in 50 µL (striatum), 100 µL (hippocampus, olfactory tubercle) or 150 µL (cortex of the cerebral hemispheres) 0.1 M hydrochloric acid (HCl). The samples were then centrifuged for 20 min with an acceleration of 14,000 × g at +6 °C. The supernatant was collected into clean plastic tubes and stored until analysis at −80 °C. The samples were rethawed on the day of analysis, after which they were centrifuged again (14,000 × g, 20 min, at +6 °C) to avoid the possible ingress of the remaining sediment particles into the chromatographic system (Karpova et al., 2016; Zhukov et al., 2022b), and analyzed at +30 °C, potential of +0.70 V, mobile phase containing 5.5 mM citrate-phosphate buffer with 0.7 mM octane sulfonic acid, 0.5 mM and 6.5% acetonitrile (pH 3.0), with the elution rate 0.8 mL/min and a 20 min analysis time (n = 11 per group, see Supplementary Table S2 in Supplementary Material).

FSCV Recordings of Dopamine Release in Rat Nucleus Accumbens (NAc) were measured in the anesthetized rats with a single intraperitoneal injection of urethane (1.5 g/kg) and fixed in a stereotaxic frame (n = 4 per group). Secretory activity of dopaminergic neurons *in vivo* was assessed by changes in the dopamine level in the intercellular space of the NAc using fast-scanning cyclic voltammetry in response to electrical stimulation of the VTA (Anstrom et al., 2009). Before the experiment, the sensory carbon microelectrode in glass insulation was calibrated *in vitro*. A stimulating electrode (bipolar steel electrode with 0.2 mm thick insulation) was implanted into the right VTA using the following coordinates relative to Bregma: AP = −5.3 mm, L = 0.8 mm, H = 8.2 mm (Paxinos and Watson, 2010). A sensory carbon microelectrode in glass insulation (open tip length 100 μm and thickness 7 μm), which records the dopamine level, was implanted ipsilaterally into the core of NAc using the following stereotaxic coordinates: AP = +2.0 mm (from Bregma); L = 1.2 mm; H = 6.8 mm from the skull surface. A reference electrode made of pressed Ag/AgCl with a 3-mm diameter was also implanted. It was located on the skull surface AP = +5.5 mm relative to Bregma; L = 0. UV-curable dental acrylic paste was used for its fixation. The electrodes were connected to a voltammetric amplifier connected to a computer running specialized software. To study the difference in the frequency dependence of dopamine release between TAAR9-KO and WT rats, VTA was stimulated with rectangular electrical pulse trains with an interval of 180 s for 1 h (current 240 μA, pulse duration 1 ms with a frequency of 100 Hz for 0.5 s). The obtained data were analyzed using the Analysis Kid web application (created by the Hashemi Lab) with calibration and data analysis tools for signals of electroactive molecules in fast-scan cyclic voltammetry (Nemets et al., 2022).

### 2.5 Behavioral assays

The Grooming Test (GT) was used to quantify both basic self-grooming metrics and grooming microstructure (n = 6–7 per group) (Kalueff et al., 2007). In brief, rats were individually placed in a transparent glass cylindrical jar (20 cm in diameter, 45 cm in height), and their grooming behavior was recorded for 10 min using an Apple iPhone 12 video camera (Apple Inc., Cupertino, United States). The recorded grooming behavior was then manually scored to determine: numbers of total grooming bouts (n), rostral grooming bouts (n), caudal grooming bouts (n), as well as grooming bouts (n) specific to the paw, nose, head, body, and tail. To further analyze grooming microstructure, the number of grooming transitions (n) between different body parts (e.g., nose to head, head to tail) was also noted. We compared the number of valid transitions within each cephalo-caudal progression and generated ethograms to represent microstructural sequences, as previously described (Kalueff et al., 2016). Any transition between grooming stages that violated the normal cephalo-caudal progression (paws → nose → head → body → tail/anogenital) was considered invalid (including skipping body parts). We also summed the numbers of seconds spent grooming each of these areas and analyzed them in terms of percentages of total grooming time. Tail and anogenital grooming (T/A) were not seen in any of the WT and TAAR9-KO rats in the present study.

The Elevated Plus Maze (EPM) was used to assess anxiety-like behavior as a highly sensitive and widely used rodent behavioral assay based on preference for protected (walled) vs. open aversive arms (n = 6 per group) (Walf and Frye, 2007). EPM was a plastic elevated maze above the floor at a height of 60 cm, featuring two open arms and two closed arms. 50-cm open arms, 30-cm closed arm with 30-cm walls, 14 cm wide. At the start of the experiment, rats were placed at the center of the EPM. Behavioral patterns (the number of grooming/hanging episodes, and total time spent in open/closed arms) were recorded by an operator over 5 min per rat, without any changes in light intensity. The anxiety index (AI) was assessed as previously described (Manukhina et al., 2021; Tseilikman et al., 2020):

$$AI=1-\frac{\left(\frac{Time\;spent\;in\;open\;arms}{\Sigma \;\text{time}\;\text{spent}\;\text{in}\;\text{maze}}\right)+\left(\frac{\#\;of\;entries\;into\;open\;arms}{\Sigma \;\text{all}\;\text{entries}}\right)}{2}$$

The Sexual Incentive Motivation Test (SIMT) was used to for the social or sexual appetitive behavior testing in male rats (n = 6 per group) (Bai et al., 2014a; Zhukov et al., 2022c). Two zones were differentiated for the analysis: a 10 × 10 cm zone closest to the stimulus cage called “female zone” and other part of cage 10 × 20 cm called “neutral zone”. The following parameters potentially descriptive of sexual behavior were analyzed manually: latency of first visit to female zone (sec), number of female zone visits (n), time in female zone (sec), time in neutral zone (sec), total time of grooming (sec), sniffing (n), stimulus-cage sniffing (n), rearing, stimulus cage wall-stand (n), fecal boli (n).

Registration of the amplitude of the acoustic startle reflex (ASR) and the index of pre-pulse inhibition (PPI). The experimental setup included a sound-attenuated chamber equipped with four floor-mounted vibration sensors, a Power1401-3A data acquisition interface (Cambridge Electronic Design, Cambridge, United Kingdom), and Spike2 software (CED). Adult rats (TAAR9-KO (n = 11) and WT (n = 12)) movement was detected by the vibration sensors, with signal amplitudes converted into millivolt values. Before the experiments, the animals were presented with white noise (74 dB) for 20 min to facilitate habituation. On the day of the experiment, each animal was presented with white noise (74 dB, 10 min), followed by 10 prepulse stimuli (78 dB, 50 ms), then by 20 pulse stimuli (100 dB, 50 ms), and finally, by 20 combined prepulse + pulse combinations (100 ms delay between stimuli). The interval between stimuli and stimulus combinations varied from 10 to 14 s. Video was recorded during the experiments in order to distinguish between spontaneous motor activity and startle responses. Prepulse inhibition index was calculated according to the following formula: PPI = (1 − (prepulse + pulse response amplitude)/pulse response amplitude) × 100%.

### 2.6 Hematological assays

Osmotic Erythrocyte Fragility Test (EFT) was performed on rat blood samples as described previously (Zhukov et al., 2020; Zhukov et al., 2021). Whole blood was collected in EDTA tubes and RBC mass was centrifuged, and sequentially resuspended in saline to purify the RBCs. Five microliters of the cell suspension were added later at room temperature to tubes containing 2.5 mL each of graded concentrations of NaCl (The latter tubes were prepared by diluting the following solution with distilled water to the appropriate salt concentration: 0.25, 0.35, 0.40, 0.45, 0.50, 0.55, 0.65%). The tubes were gently mixed again and after standing 10 min the unlysed RBCs were removed by centrifugation. The relative amount of hemoglobin released into the supernatant was measured spectrophotometrically at 541, 555, and 577 nm, using a 0.85% NaCl sample as the blank and a 0.1% sample as the 100% lysis point.

For other hematological measurements, we used the automatic analyzer Advia Centaur XP (Siemens Healthcare Diagnostics, Germany), which automates standardized enzyme-linked immunosorbent assays (ELISAs) with chemiluminescent quantification. The hormones measured were testosterone, progesterone, TSH, FT3, and FT4; antibodies for these are provided by the analyzer’s manufacturer (Siemens Healthcare Diagnostics, Germany). Serum samples were diluted with sterile pyrogen-free 0.9% sodium chloride solution in a ratio of 1:6. Prothrombin time, activated partial thromboplastin time, fibrinogen content assays were conducted by an automatic coagulation analyzer CoaLab 1000 (LABiTec, Germany). We also analyzed electrolyte and blood gas levels using Siemens RapidLab 1265 (Siemens Healthcare Diagnostics, Germany). The Arterial Blood Pressure (ABP) was measured using a noninvasive blood pressure monitor NIBP-8 (Columbus Instruments, Columbus, OH) (n = 7–12 per group).

### 2.7 Sample collection and storage methods

Sampling blood from the lateral tail vein of the rat was used in hematological experiments as was described before (Lee and Goosens, 2015). Whole blood was used for the erythrocyte fragility test (EFT) and electrolytes assays. The samples were placed into VACUETTE K3-EDTA tubes (Greiner Bio-One, Austria) solution and analyzed within 1–3 h after blood collection. The collected whole blood specimens were stored at +4 °C or at room temperature before the experiment. After the complete blood count (CBC) measurement, red blood cells from each sample were isolated for the EFT *via* centrifugation. Serum for automated ELISA was collected in clean plastic VACUETTE blood collection tubes (Greiner Bio-One, Austria), left in a vertical position for 15 min, and then transferred to +4 °C in the same position. Samples with coagulated blood were centrifuged at 2,000 × g for 15 min at +4 °C. The serum was collected using a pipette dispenser into dry, clean tubes and stored at −20 °C for no more than 5 days until analysis. Plasma was used for coagulation analysis. Blood was collected in Vacuette Blood Sample Tube Blue (Greiner Bio-One, Austria) containing the buffered anticoagulant sodium citrate solution.

### 2.8 Inter-assay repeatability

Before analyzing the serum and blood samples, equipment was decontaminated, calibrated, and checked by internal quality control. Inter-assay repeatability was evaluated by calculating the coefficient of variation (CV) of 10 consecutive measurements of internal quality control material in three different controls (low, normal, and high). Coefficients of variation (CV) were computed as 100% times the ratio of the standard deviation *σ* to the mean *μ* (i.e., CV = (σ/μ) × 100%)

### 2.9 Statistical analysis and visualization

Some data were analyzed using the Wilcoxon-Mann–Whitney U-test, using GraphPad Prism version 6.0 for Windows (GraphPad Software, United States). This test is non-parametric in that it compares rank sums instead of means. The unpaired t-test was applied to compare means and measures of spread whenever its assumptions were satisfied. Values of p < 0.05 were considered to be significant in both cases. Each figure caption in the article reports the test applied to the respective datasets. Additional visualizations were conducted with a licensed account on BioRender.com.

## 3 Results

### 3.1 Rat brainstem and midbrain express *Taar9* mRNA

**FIGURE 2:**
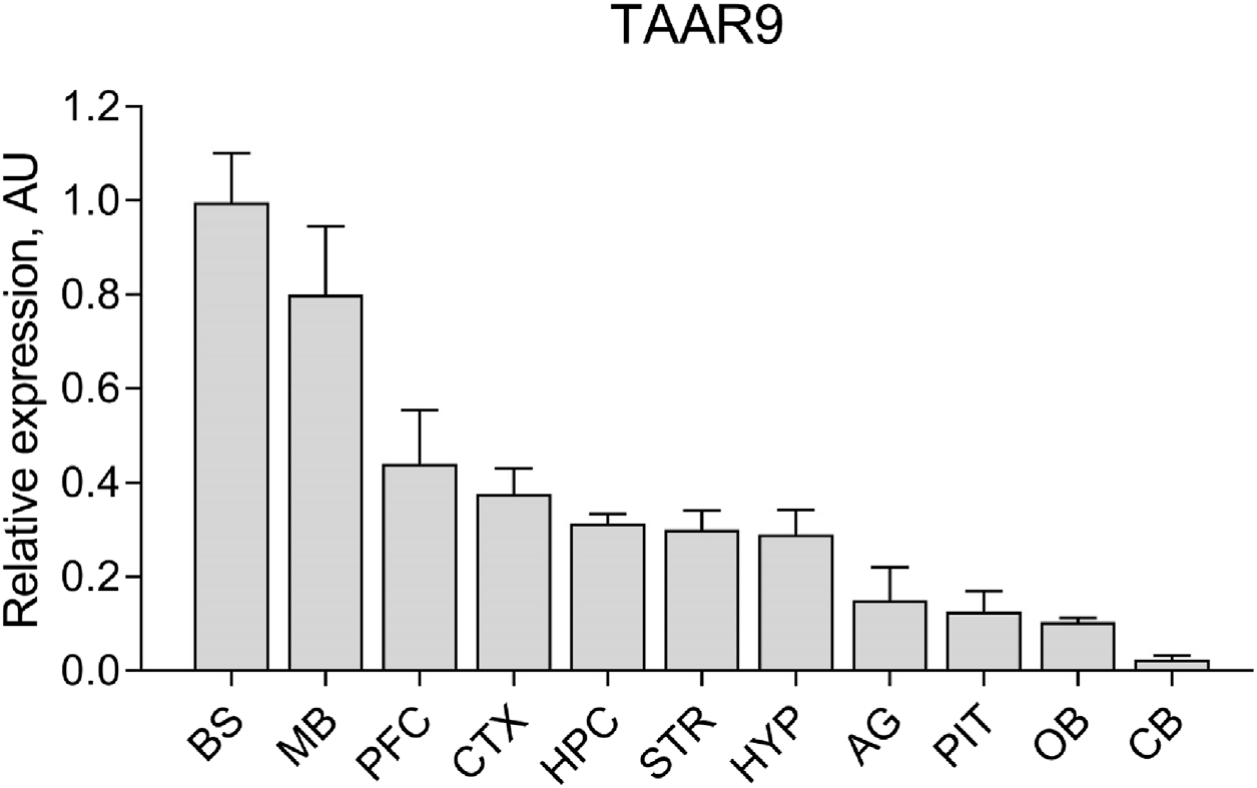
Relative expression levels of *Taar9* in rat brain and adrenal gland samples shown in arbitrary units (AU). The mRNA expression levels of *Taar9* were measured by qPCR in tissues of Sprague-Dawley (SD) rats and normalized to the expression of the housekeeping gene TATA-box binding protein (TBP) on a per sample basis (n = 3 wild-type SD rats). BS, brainstem; MB, midbrain; PFC, prefrontal cortex; CTX, cortex; HPC, hippocampus; STR, striatum; HYP, hypothalamus; AG, adrenal glands; PIT, pituitary gland; OB, olfactory bulb; CB, cerebellum.

*Taar9* mRNA expression was detected in various brain regions of Sprague-Dawley (SD) rats. Although *Taar9* expression appeared low across all tissues examined, the highest levels were observed in the brainstem and midbrain. Lower levels were detected in the cortex, prefrontal cortex, hippocampus, striatum, hypothalamus, olfactory bulb, as well as in the pituitary and adrenal glands (Figure 2).

These data indicate that the rat brain is a key site of TAAR9 activity. The elevated expression of TAAR9 in the midbrain and brainstem, which contain the nuclei of dopaminergic neurons (substantia nigra, ventral tegmental area), serotonergic neurons (raphe nuclei), and noradrenergic neurons (locus coeruleus) suggests a potential role for TAAR9 in modulating monoaminergic activity. This observation prompted us to conduct a broad characterization of TAAR9-KO rats, including both biochemical and behavioral assays.

### 3.2 *Taar9* deletion affects hippocampal serotonin levels, and dopamine turnover, but not dopamine release in the mesolimbic pathway

**FIGURE 3:**
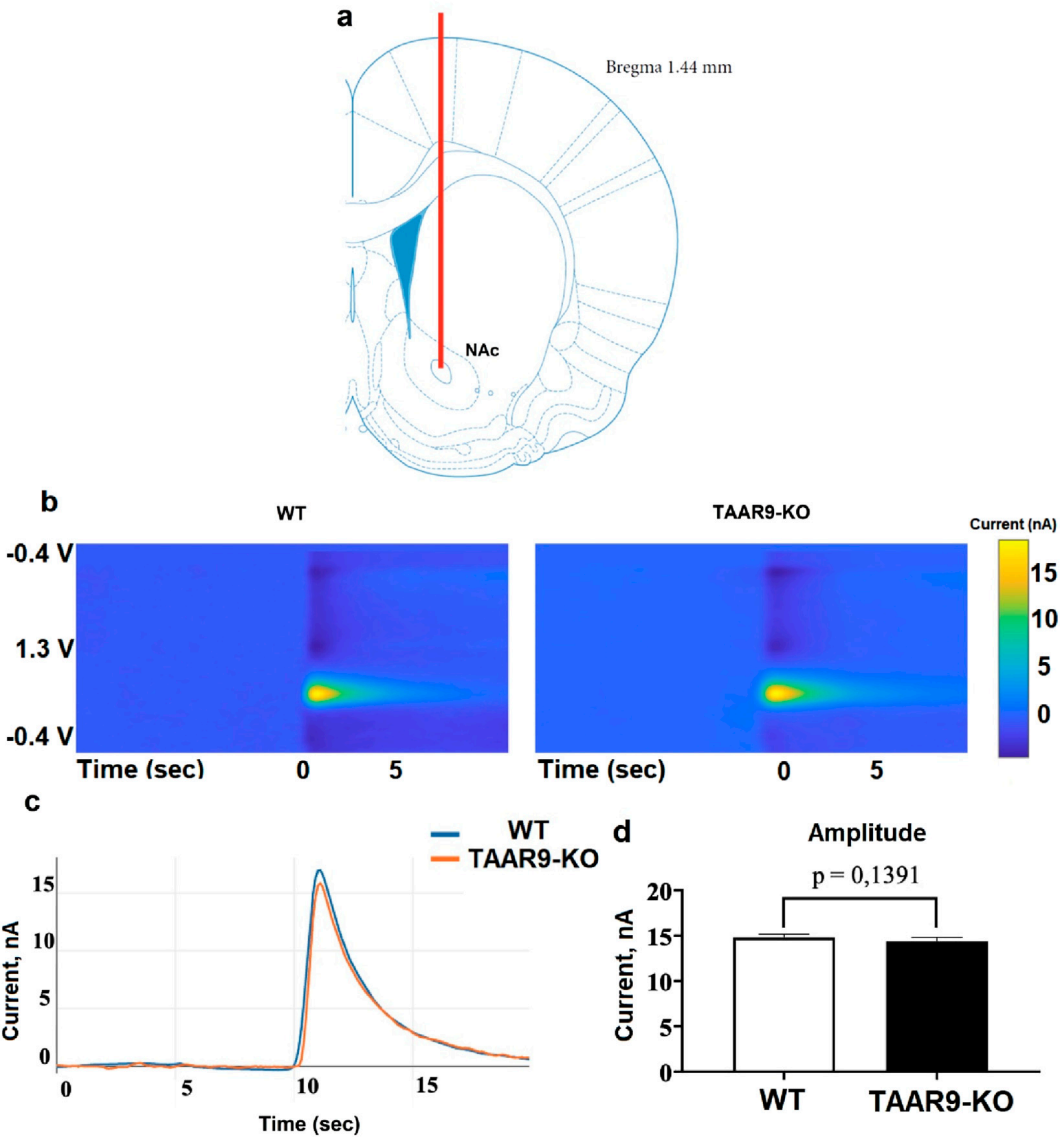
TAAR9 knockout does not affect induced mesolimbic dopamine release in rats. Using fast-scanning cyclic voltammetry (FSCV), the secretory activity of dopaminergic neurons was assessed in WT and TAAR9-KO rats based on the change in the level of dopamine in the intercellular space of the nucleus accumbens (NAc) in response to electrical stimulation of the ventral tegmental area (VTA) *in vivo*. **(a)** shows the position of the measuring electrode in the core region of NAc, **(b)** depicts representative color plots of the redox potentials which signify current in nanoamperes (nA) at the given voltages (V) across time in seconds (sec), **(c)** shows the mean signals of these color plots (n = 4 per genotype), and **(d)** reports the mean peaks ±SEM of these signals; the difference between these peaks was not significant as per the Mann–Whitney U-test (p > 0.05).

Fast-scan cyclic voltammetry (FSCV) was used to measure the mesolimbic release of dopaminergic neurons in WT and TAAR9-KO groups. Specifically, changes in dopamine levels in the extracellular space of the nucleus accumbens (NAc) were monitored in response to electrical stimulation of the ventral tegmental area (VTA) *in vivo*. Knockout of TAAR9 did not affect the magnitude of the induced dopamine release at the NAc (Figures 3a–d).

**FIGURE 4:**
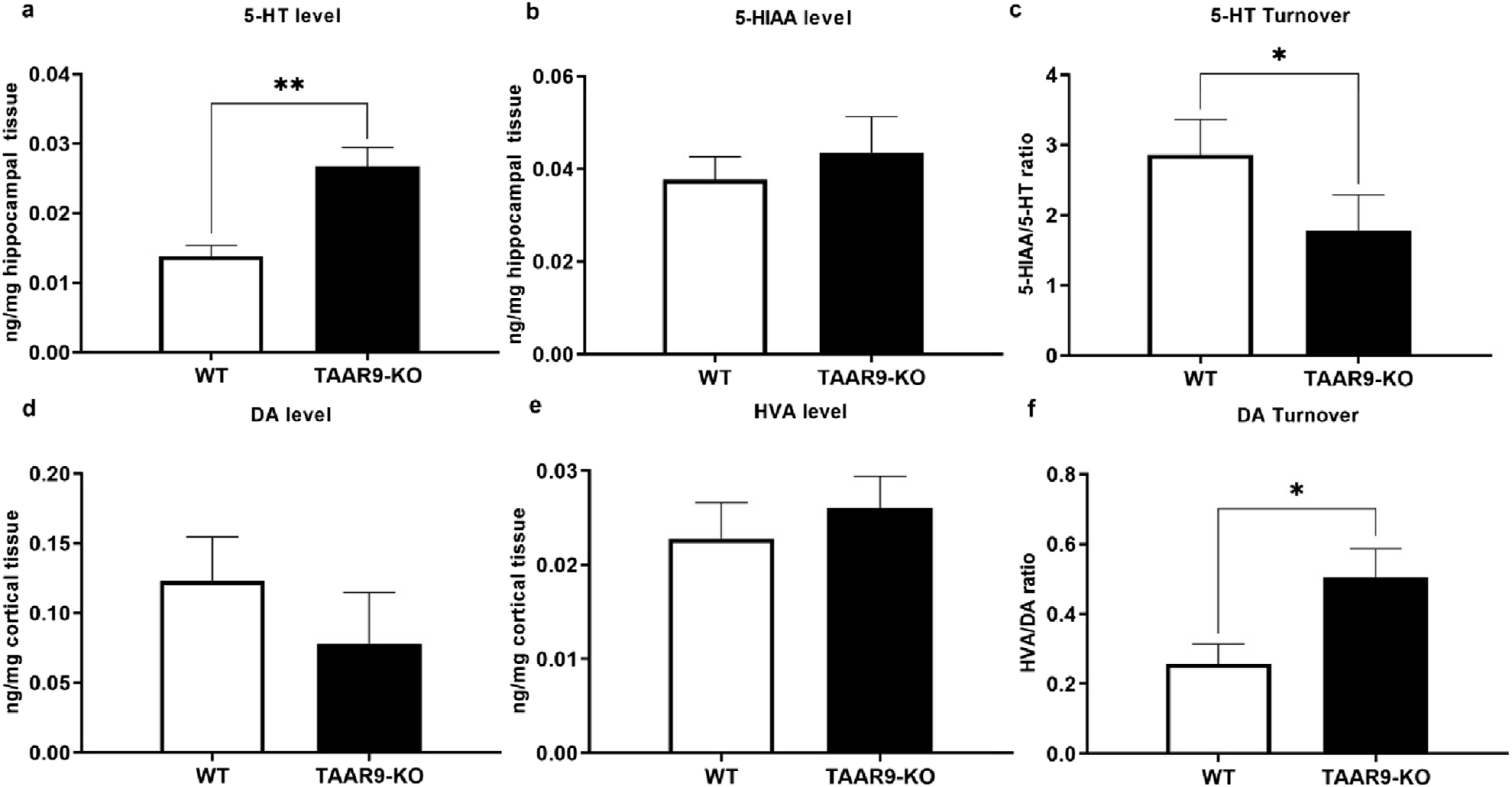
TAAR9 knock out alters the levels of monoamines and their metabolites in the rat brain. Hippocampal levels of 5-HT (p = 0.0030) and turnover (5-HIAA/5-HT) ratio (p = 0.0350) significantly differed across genotypes **(a–c)**, whereas changes in the cortical levels of DA and HVA did not reach significance **(d,e)**, despite a significant change in cortical dopamine turnover **(f)** (HVA/DA ratio, p = 0.0328). All data are presented as mean ± SEM (n = 11 per group). * p < 0.05, ** p < 0.005, Mann–Whitney U-test.

As shown in Figure 4, hippocampal 5-HT levels were significantly higher in TAAR9-KO rats compared to controls (Figure 4a; p = 0.0030). In contrast, 5-hydroxyindoleacetic acid (5-HIAA) levels in hippocampus remained unaltered (Figure 4b). As such, the 5-HT turnover rate, expressed as the 5-HIAA/5-HT ratio, was significantly lower in mutant hippocampi (Figure 4c; p = 0.0350). Additionally, a significant increase was observed in the homovanillic acid/dopamine (HVA/DA) ratio (p = 0.0328) (Figures 4f). Other neurochemical parameters across different brain structures remained unaffected (Supplementary Table S1).

### 3.3 *Taar9* deletion is sufficient to alter grooming, but not other behaviors

**FIGURE 5:**
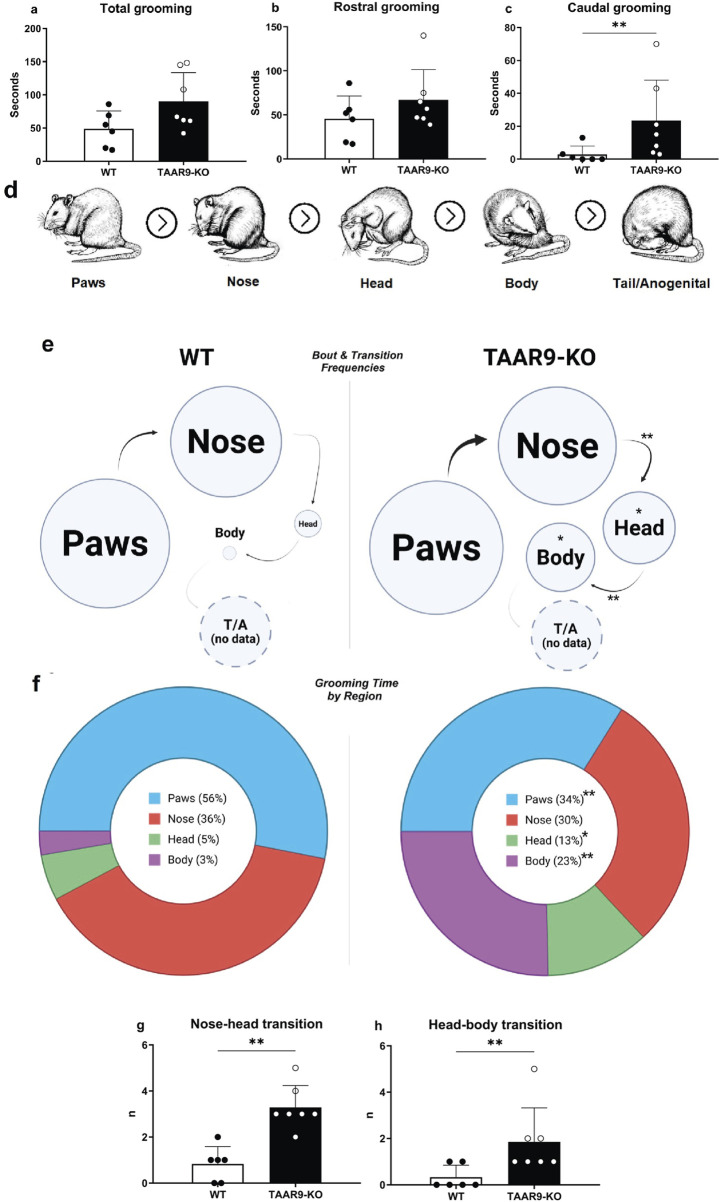
Comparative grooming microstructure analysis of TAAR9-KO vs. WT rats (n = 6-7 per group). Total grooming **(a)** and rostral grooming **(b)** durations did not significantly differ in the two genotypes, whereas caudal grooming duration **(c)** increased (p = 0.0093) in the mutants; rostral and caudal were defined with respect to the forelimbs in this breakdown. **(d)** shows visual definitions of region-specific grooming bouts in the evolutionarily conserved cephalocaudal order. Ethograms **(e)** depict regional biases in self-grooming based on these definitions, where circle diameter and arrow thickness reflect the mean frequencies of region-specific grooming bouts and of transitions, respectively. All grooming bouts, but only transitions conforming to the cephalocaudal order were statistically assessed **(d)**. The frequencies of nose → head (p = 0.0017) and head → body (p = 0.0087) transitions significantly increased in the TAAR9-KO group **(g,h)**, as well as the mean frequencies of head (p = 0.0105) and body (p = 0.0204) grooming bouts **(e)**. Pie-charts **(f)** depict the percentages of total grooming time spent per region, irrespective of whether the bouts conformed to the cephalocaudal order paws (p = 0.0012), head (p = 0.0338), body (p = 0.0047). No tail/anogenital grooming (T/A) occurred in any of the 10-min video recordings. Full results are presented in Supplementary Table S1. * p < 0.05, ** p < 0.005, Mann–Whitney U-test.

As shown in Figure 5, significant alterations were revealed in the self-grooming endpoints, as the number of nose-head (p = 0.0004) and head-body (p = 0.0087) transitions significantly increased in the TAAR9-KO group, as did as the total number of head- (p = 0.0105) and body-directed (p = 0.0204) grooming bouts and time spent grooming the body (p = 0.0047).

**FIGURE 6:**
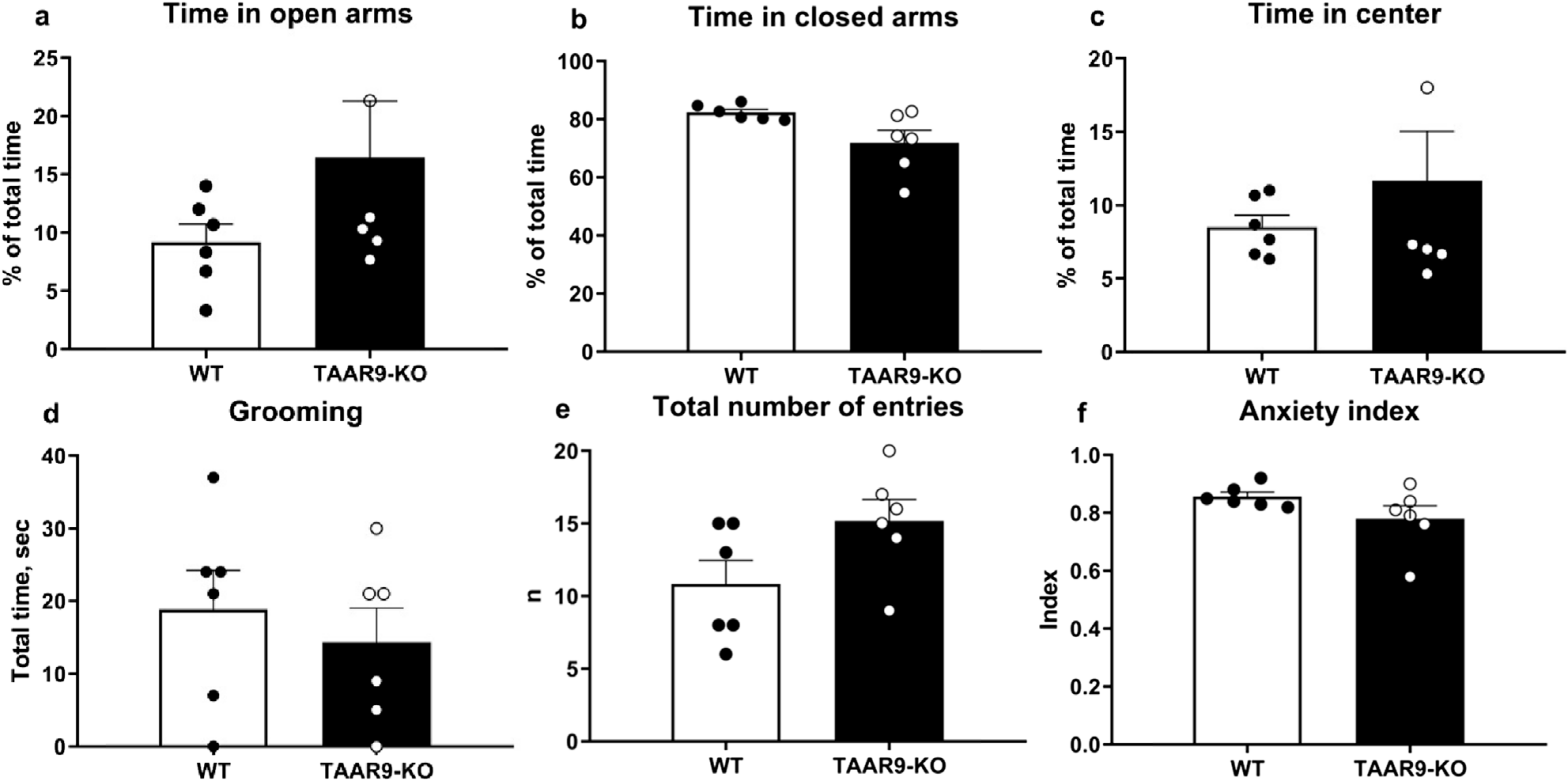
Elevated Plus Maze (EPM) evaluation of anxiety-like behaviors in TAAR9-KO vs. WT rats, depicting **(a)** % of time in open arms, **(b)** % of time in closed arms, **(c)** % of time in the center, **(d)** time spent grooming, **(e)** total number of entries and **(f)** the anxiety index. No significant differences arose as per the Mann-Whitney U test (p > 0.05). All graphs show means ± SEM (n = 6 per group).

**FIGURE 7:**
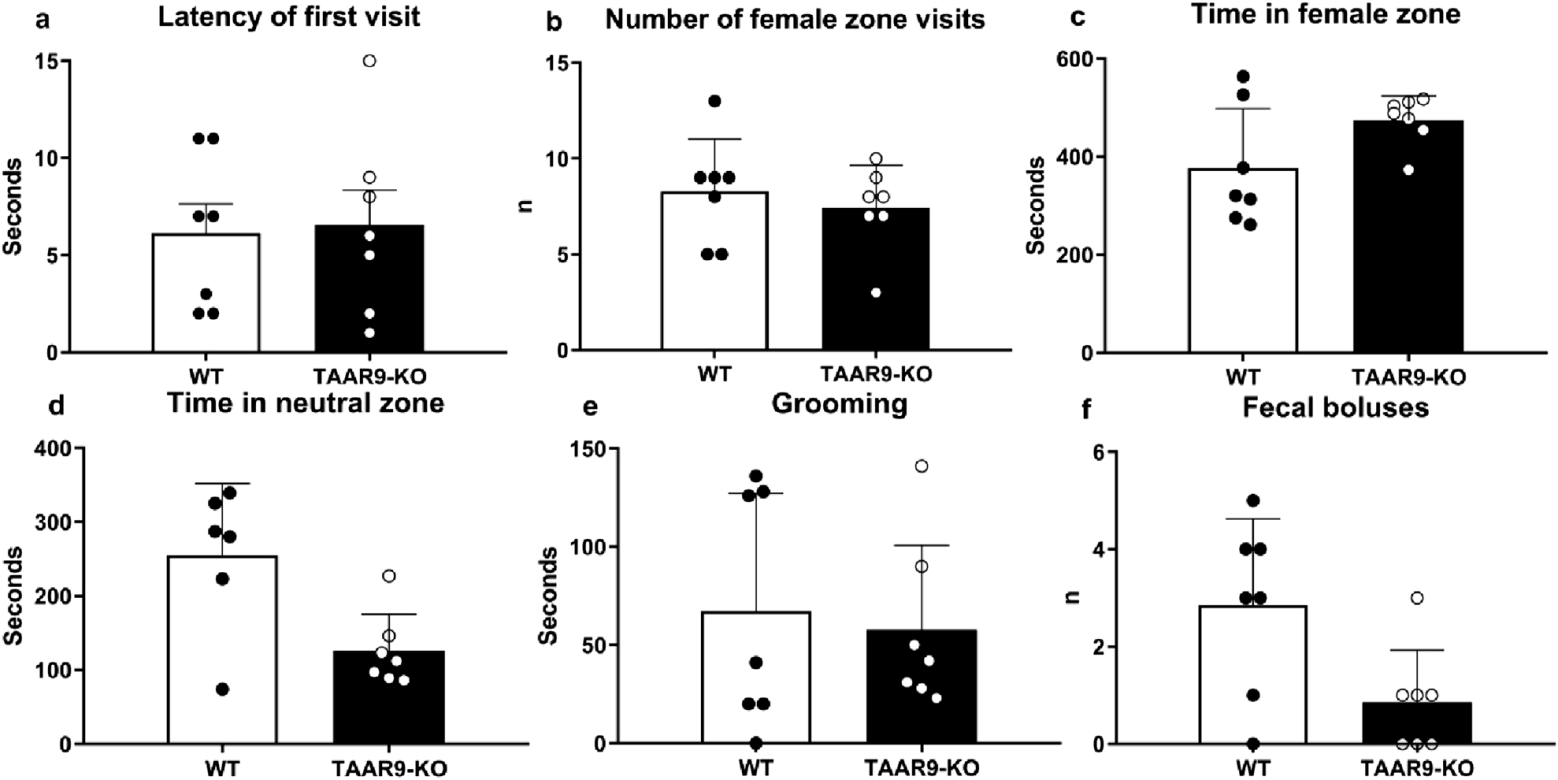
Sexual Incentive Motivation Test (SIMT) evaluation of sexual motivation in TAAR9-KO vs. WT rats. No significant changes (p > 0.05) were found in the **(a)** latency of first visit to female zone, **(b)** number of female zone visits, **(c)** time in female zone, **(d)** time in neutral zone, **(e)** total time of grooming, and **(f)** number of fecal boluses. Data are presented as mean ± SEM (n = 11 per group); all comparisons were made by the Mann-Whitney U test.

The exploratory and anxiety-like behaviors of TAAR9-KO rats were evaluated in the elevated-plus maze (EPM). Genotype exerted no significant effects on any readouts in the EPM (Figure 6.), despite the mutants showing a consistent and qualitative increase in within-group variance (Figures 6a–c,e). The same was true of all metrics in the sexual incentive motivation test (SIMT) (Figure 7).

**FIGURE 8:**
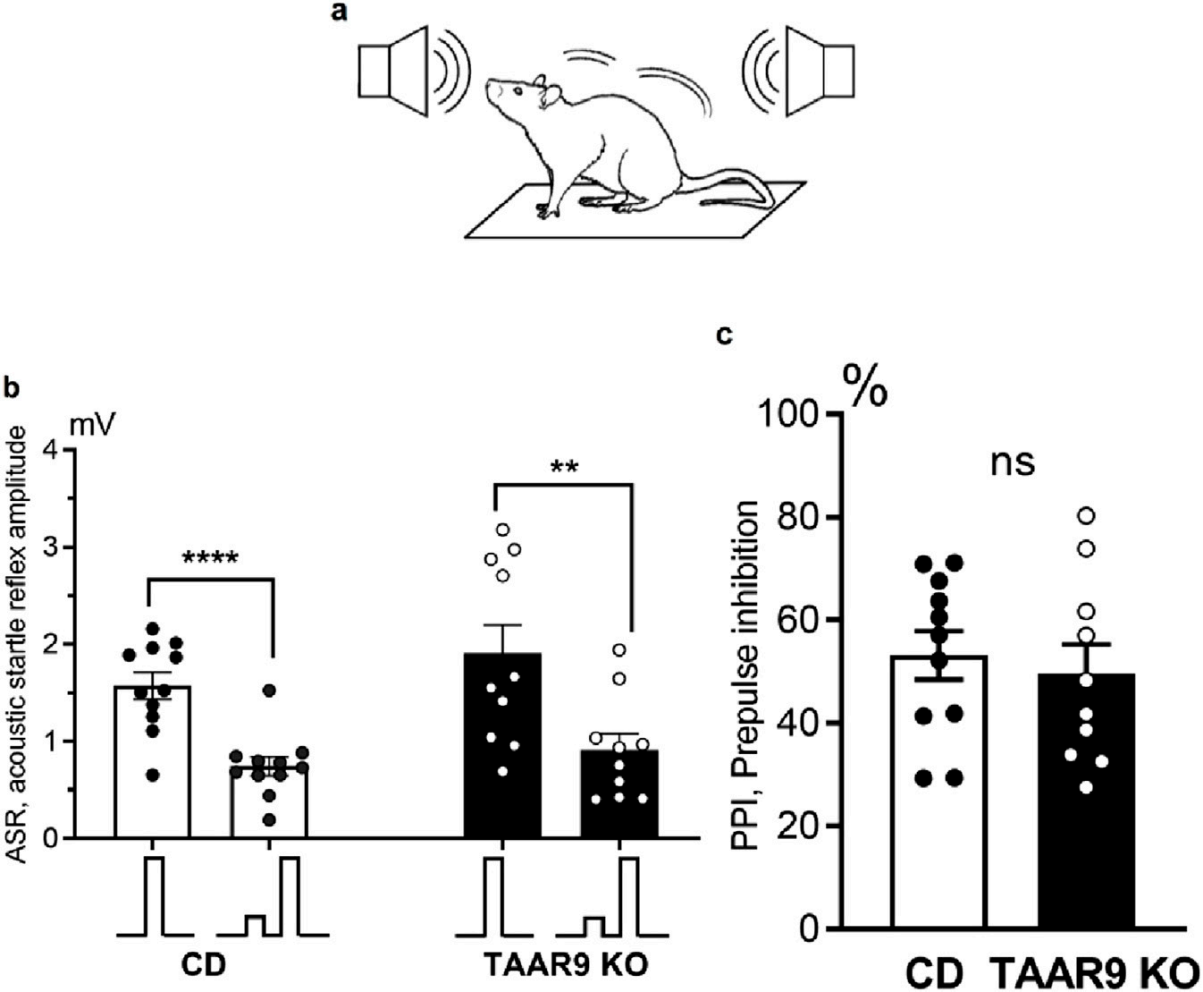
Prepulse inhibition (PPI) of the acoustic startle reflex (ASR) compared across genotypes. **(a)** depicts the set-up, wherein vibration sensors installed on the platform floor were used to record animal movements in response to acoustic stimulation, thus operationalizing the ASR as an amplitude in millivolts (mV); **(b)** shows these ASR amplitudes, with the inset below depicting the relative tone volumes of the single and paired pulses. Prepulses significantly inhibited the ASR to the full-volume pulses in both genotypes **(b)**, and the PPI index (i.e., percent decrease in ASR after prepulse) **(c)** was the same in TAAR9-KO and WT rats. All graphs show means ± SEM (n = 11–12 per group); ** p < 0.01; **** p < 0.0001; ns - not significant; unpaired t-test.

The amplitudes of ASRs and the PPI index were measured in TAAR9-KO and WT rats. Analysis of the results showed that the amplitude of the ASR to the pulse alone was significantly greater than the response to the combined prepulse + pulse acoustic stimulus (Figure 8). This applies to both TAAR9-KO rats (paired t-test, p = 0.0014) and WT control rats (paired t-test, p < 0.0001). However, there was no significant difference in the amplitude of the ASR between TAAR9-KO rats and WT controls. Comparison of PPI index values between TAAR9-KO (53.19 ± 4.69) and WT (49.56 ± 5.72) rats also revealed no significant differences (unpaired t-test, p = 0.63) (Figure 6c).

### 3.4 Extended hematological assays show no aberrations in TAAR9-KO rats

We investigated if the deletion of the TAAR9 gene affects hematological parameters such as erythrocyte fragility, hormone levels, blood pressure, coagulatory parameters, and electrolyte levels.

**FIGURE 9:**
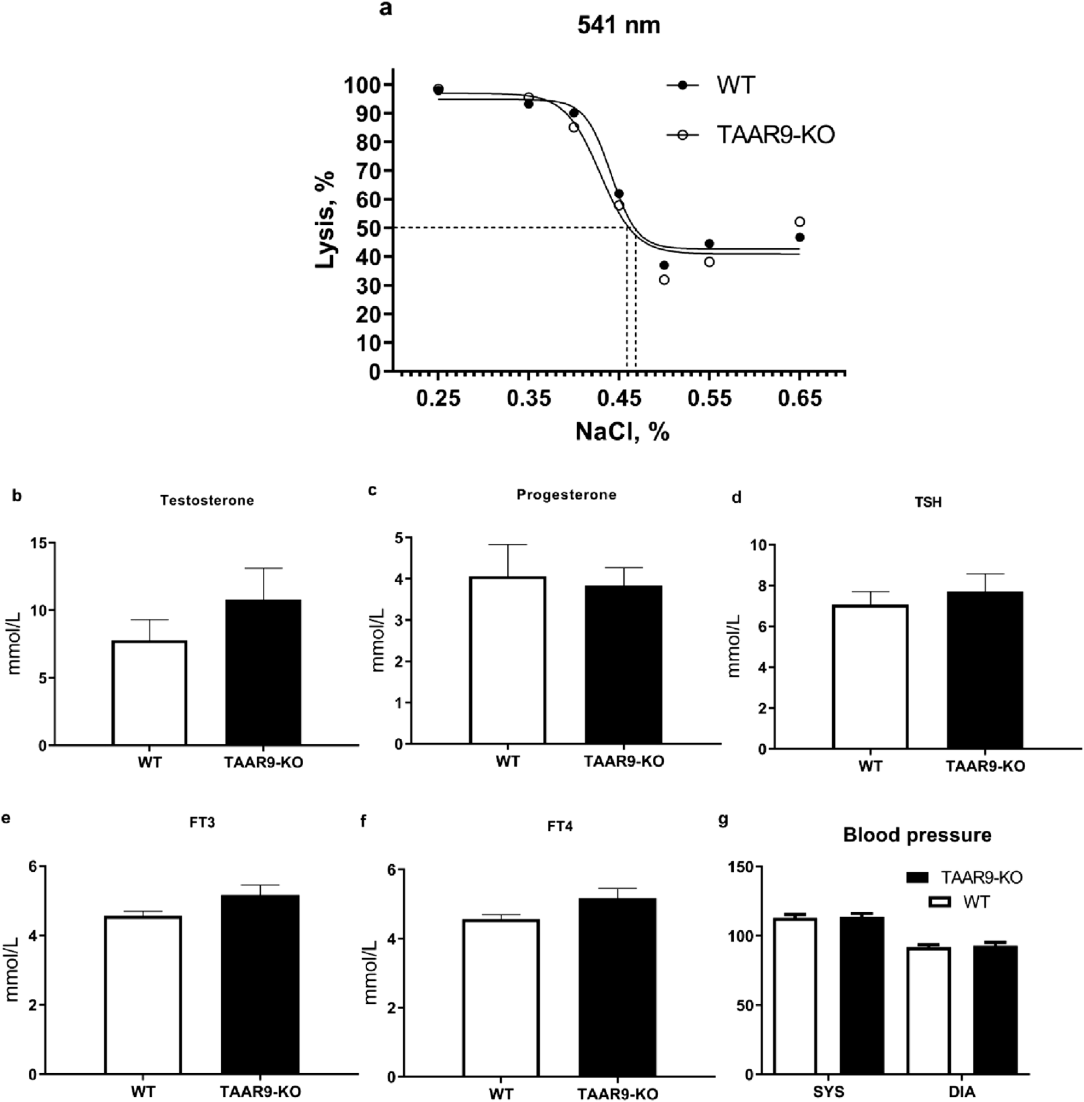
Comparison of hematological parameters from WT and TAAR9-KO rats. **(a)** shows a representative graph of averaged spectrophotometric measurements quantifying erythrocyte fragility by % lysis at increasing salt concentrations plotted as % (w/v) salt; the plotted 50% lysis points (dashed lines) were WT = 0.468 and KO = 0.459% salt. Graphs **(b–f)** depict an automatic blood analyzer’s estimates of millimoles hormone per liter serum. Graph **(g)** shows systolic (SYS) and diastolic (DIA) arterial pressure in mmHg measured in rat tails. Hormone abbreviations: TSH, thyroid stimulating hormone; FT3, free triiodothyronine; FT4, free thyroxine. All reported data are means (n = 9–10 per group) and error bars depict respective SEM values. All comparisons were not significant as per the unpaired t-test (p > 0.05).

In previous studies on two strains of TAAR9-KO rats (insA/delC) (Murtazina et al., 2021), we found mixed results in the electrolyte fragility test (EFT). In the present study, we used TAAR9-KOdelC rats, backcrossed to the SD background for six generations to validate alterations in erythrocyte pathologies. The relative amount of hemoglobin released into the supernatant was determined spectrophotometrically at 541, 555, and 577 nm, with different concentrations of NaCI. Measurement at multiple wavelengths allows for the detection of differences in the fractional absorption of oxyhemoglobin and deoxyhemoglobin (Dewey et al., 1982). No differences in fractional absorption were observed, as absorption spectra were almost identical at all three wavelengths. All these data are available in Supplementary Figure S1. Representative results at 541 nm are shown in Figure 9a.

To exclude the possibility that the observed behavioral alterations were caused by hormonal imbalances in TAAR9-KO rats, we measured several key hormonal parameters. Genetic deletion of this gene did not affect levels of testosterone, progesterone, thyroid stimulating hormone (TSH), free triiodothyronine (FT3), or free thyroxine (FT4) (Figures 9b–f). Furthermore, we assessed cardiovascular function by measuring arterial pressure at the tail, which also remained unaltered in TAAR9-KO rats (Figure 9g). All coagulator and blood electrolyte parameters were normal in TAAR9-KO rats (Table 1).

**TABLE 1:** Comparison of blood coagulatory and electrolyte parameters in TAAR9-KO vs. WT rats. Data are presented as mean ± SEM. All comparisons were not significant as per the unpaired t-test (p > 0.05).

Blood parameter	WT	TAAR9-KO	N ^WT/KO^	P value
FibDS, g/L FibDS, sec PT-DS, % PT-DS, sec PT-DS, INR	3.08 ± 0.21 9.52 ± 1.43 59.34 ± 2.08 15.13 ± 0.26 1.35 ± 0.03	3.76 ± 0.46 7.83 ± 0.87 64.74 ± 2.47 14.51 ± 0.25 1.29 ± 0.03	9/9 9/9 9/9 9/9 9/9	NS (0.203) NS (0.331) NS (0.114) NS (0.102) NS (0.097)
APTDS, sec pH pCO2, mmHg pO2, mmHg Na, mmol/L	24.77 ± 0.81 7.22 ± 0.06 45.84 ± 4.65 155.77 ± 10.57 143.41 ± 2.68	26.66 ± 0.77 7.25 ± 0.06 47.29 ± 5.21 160.38 ± 10.60 146.70 ± 2.38	9/9 7/12 7/12 7/12 7/12	NS (0.112) NS (0.722) NS (0.819) NS (0.746) NS (0.189)
K+, mmol/L Cl-, mmol/L HCO3-act, mmol/L O2SAT(est), %	9.97 ± 1.02 82.00 ± 3.52 17.91 ± 1.23 98.20 ± 0.41	9.19 ± 1.15 89.17 ± 2.06 20.21 ± 1.25 98.17 ± 0.39	7/12 7/12 7/12 7/12	NS (0.592) NS (0.108) NS (0.361) NS (0.735)

Abbreviations: KO, knockout; WT, wild-type; FibDS, fibrin degradation products; PT-DS, prothrombin time degradation products; APTDS, activated partial thromboplastin time.

## 4 Discussion

The present study demonstrates for the first time that TAAR9 plays a significant regulatory role in the monoaminergic systems of the brain. Measurements in TAAR9-KO rats revealed increased hippocampal serotonin and altered dopamine turnover in the cerebral cortex (Figure 4). These effects make sense given the relative enrichment of the receptor in the brainstem and its lesser expression in the midbrain (Figure 2). Deep within the brainstem, serotonergic dorsal raphe nuclei (DRN) and the noradrenergic locus coeruleus innervate much of the cortex, and several subcortical structures such as the hippocampus and amygdala (Foote et al., 1983; Liebe et al., 2022; Manger and Eschenko, 2021). The midbrain contains the VTA, which is the chief dopaminergic and glutamatergic node of the mesolimbic and mesocortical pathways (Cai and Tong, 2022; Pezze and Feldon, 2004; Yamaguchi et al., 2011). Consistent with the relative expression data, the impact of TAAR9 is more pronounced on 5-HT than it is on DA. The DA system is critical to PPI and our results concordantly show negligible effects in both (Figures 4, 8). Indeed, ablation of TAAR9 exerts no effect on the mesolimbic pathway, as *in vivo* FSCV showed unaltered DA release in the NAc upon VTA stimulation (Figure 3). This is despite a significant increase in what appears to be DA catabolism (Figure 4f). There have been commendable strides in the study of TAAR9 as an olfactory receptor (Guo et al., 2023; Q. Li et al., 2015; Q. Li and Liberles, 2016; Liberles, 2015), but this study is the first to show an independent role for TAAR9 in the monoaminergic nuclei of the brainstem.

### 4.1 Perturbed olfaction in TAAR9-KO rats is behaviorally relevant

The observed changes in self-grooming patterns demonstrate that the functions of TAAR9 are behaviorally significant, and a conventional interpretation of the grooming bias in TAAR9-KO rats implies an analogy with obsessive-compulsive disorder (Shmelkov et al., 2010; Yang and Lu, 2011; Zike et al., 2017). A strong case has been made that certain grooming biases in rodents indicate phenotypes analogous to a variety of psychiatric disorders (Kalueff et al., 2007). That may be true in general, but the olfactory functions of TAAR9, and the critical importance of olfaction in rodent behavior likely obfuscate the meanings of such grooming biases. For instance, a grooming bias towards the body (i.e., areas posterior to the forelimbs and anterior to the anogenital segment) has been associated with an increased overall duration of grooming and anxiety-like behaviors in the EPM (Kalueff et al., 2007; Liu et al., 2021). Those two correlates seem reversed in our dataset–TAAR9-KO rats seemed to exhibit no change in the durations of grooming, and floor effects in the EPM, despite a significant grooming bias towards the body. This is a case wherein the interpretation of grooming results is simply not reliable. Indeed, guidelines list many caveats in drawing specific conclusions from grooming data (Kalueff et al., 2007). Grooming analysis is nevertheless sensitive to neurological changes, and in this study, it implied that the observed shift in hippocampal serotonin was sufficient for behavior change–a simple proof of concept.

Given the influence of olfactory cues on mammalian sexuality, we were interested to see whether the information encoded by TAAR9 was sexually relevant. The SIMT allows for the assessment of male rodent sexual motivation driven by female estrous secretions of volatile compounds (Bai et al., 2014b; Eliasson and Meyerson, 1975). In previous studies, we observed no changes in TAAR1 knockout mice (Zhukov et al., 2022b) and only subtle effects in TAAR2 and TAAR5 knock out mice (Ptukha et al., 2021). This is in line with the fact that TAAR1 is the sole member of the TAAR family that does not function as an olfactory receptor in mammals (Liberles and Buck, 2006). Here, TAAR9-KO rats exhibited unaltered non-contact sexual motivation parameters (Figure 7). Despite these negative results, the idea that the TAARs can influence sexual behavior through olfactory cues is still plausible (Harmeier et al., 2018) (see reference (Li and Liberles, 2015) for relevant ethology), especially since no interventions besides *Taar* deletion were applied in the aforementioned results. Human vaginal secretions contain volatile amines which the TAARs can detect (Harmeier et al., 2018; Liberles, 2015; Nelson et al., 2015), and sex differences in the levels of amino acids exist in human sweat (Dunstan et al., 2017). Such signals could plausibly exert subtle and interesting effects on variables such as mate-choice, or even the activation of vestigial sperm-competition mechanisms that affect fertility. A case in point is cadaverine–a volatile TAAR9 agonist present in both vaginal fluid and semen (Liberles, 2015; Nelson et al., 2015; Rodríguez-Páez et al., 2023).

### 4.2 Peripheral effects of TAAR9 signaling

Our previous work on TAAR9-KO rats had confirmed two robust peripheral effects of *Taar9* deletion: 1) lower cholesterol levels (Murtazina et al., 2021), and 2) increased saccharibacteria (a.k.a. TM7) in the gut microbiota (Zhukov et al., 2022c). It is still difficult to infer a physiological mechanism that can account for such changes, and one of our main goals with the TAARs is to define their overall functions in physiology. To this end, we assessed some basic parameters and hormone levels in sera. The hematological assays showed no changes in peripheral sex and thyroid hormone levels; these results should help restrict the possibility space of future models. With respect to 5-HT, the DRN can affect the activity of the locus coeruleus in the brainstem (Lee, 2004; Pudovkina et al., 2002). Serotonin and norepinephrine also regulate each other’s levels in different brain regions (Agster et al., 2013; Fitzgerald, 2012; Gianni and Pasqualetti, 2023; Özçete et al., 2024; Vizi and Kiss, 1998). Furthermore, it is widely understood that peripheral norepinephrine regulates vasoconstriction, and thus blood pressure, but results have been mixed on whether to the locus coeruleus can directly affect these variables (Anselmo-Franci et al., 1999; Murase et al., 1994). The rats in the present study showed no change in blood pressure. Overall, the current understanding of TAAR9’s functions in the periphery is limited, and these data may aid future work in defining the relevant physiological mechanisms. Human TAAR9 transcripts have thus far been detected in the intestinal tract, and lymphocytes in blood (Berry et al., 2017). With respect to the brain, perhaps the most interesting peripheral effects of TAAR9 are the altered TM7 and cholesterol levels, which seem to correlate with relevant endpoints (Bor et al., 2019; Fan et al., 2023; Fiedorowicz and Haynes, 2010; Li et al., 2022; Russell et al., 2019; Tall and Yvan-Charvet, 2015).

## 5 Conclusion

Overall, the deletion of *Taar9* had little effect on most behavioral results, despite a significant effect on grooming behavior. The expression of *Taar9* in the brainstem, and the increased level of serotonin in the hippocampus, nevertheless warrant further investigations on the role of TAAR9 in the brain. The appeal of the TAARs is especially clear with respect to hippocampal neurogenesis and anxiety (Alnefeesi et al., 2024), since three of the TAARs have already been demonstrated to regulate this neurogenic niche (Efimova et al., 2021; 2022; Zhang et al., 2024), and strong evidence links hippocampal serotonin and plasticity to anxiolysis (Bannerman et al., 2003; Hill et al., 2015; Karayol et al., 2021; Lee et al., 2012; 2016; Shi et al., 2023; Wei et al., 2024). In this light, future investigations will have to include anxiogenic manipulations in knockout animals to test the possible significance of TAAR9, and the TAARs more generally, in anxiety.

## Data Availability

The datasets presented in this study can be found in online repositories. The names of the repository/repositories and accession number(s) can be found in the article/Supplementary Material.
