# Supplementary material for: TAAR9 knockout increases hippocampal serotonin and alters grooming behavior in rats
Source: Front Pharmacol. 2025 Nov 12;16:1684029. doi: 10.3389/fphar.2025.1684029 (PMC12647061; doi:10.3389/fphar.2025.1684029)
Supplement: Supplementary file 1 [file DataSheet1.pdf]

Supplementary Table 1. Summary of statistical results of TAAR9 knockout-induced behavioral changes in mouse self-grooming test and self-grooming microstructure analysis (n = 6-7).

**Endpoint**	**WT**	**TAAR9-KO**	**U**	***p* Value**
Total grooming bouts, n	10.5 ± 22.3	15 ± 2.12	11	0.0165
Rostral grooming bouts, n	10 ± 2.3	12.14 ± 1.73	15.5	0.4668
Caudal grooming bouts, n	0.5 ± 0.2	2.86 ± 0.77 *	4.5	0.0204
Paws grooming bouts, n	4.83 ± 1.103	5 ± 0.79	18.5	0.7558
Nose grooming bouts, n	4.17 ± 0.792	4.43 ± 0.75	19.5	0.8601
Head grooming bouts, n	1 ± 3.651	2.71 ± 0.36 *	4	0.0105
Body grooming bouts, n	0.5 ± 0.223	2.57 ± 0.685 *	4.5	0.0204
Paws to nose transitions, n	3.3 ± 0.54	6.43 ± 1.48	10	0.1300
Nose to head transitions, n	0.83 ± 0.31	3.29 ± 0.36 **	0.5	0.0017
Head to body transitions, n	0.33 ± 0.21	1.86 ± 0.55 **	4	0.0087
Paws grooming duration, sec	25.83 ± 4.18	30.43 ± 6.72	20	0.9452
Nose grooming duration, sec	19 ± 4.14	26.14 ± 6.15	17	0.5967
Head grooming duration, sec	2.5 ± 0.91	10.42 ± 1.27 **	1.5	0.0029
Body grooming duration, sec	1.3 ± 0.58	22.71 ± 8.75 **	2	0.0047
Paws grooming duration, %	55.8 ± 2.9	34.36 ± 3.18**	0	0.0012
Nose grooming duration, %	36.29 ± 2.9	29.48 ± 3.51	11	0.1807
Head grooming duration, %	5.19 ± 2.19	12.91 ± 1.73 *	6	0.0338
Body grooming duration, %	2.7 ± 1.13	22.75 ± 6.29 **	2	0.0047

Data are presented as mean ± SEM. *p<0.05, **p<0.005 TAAR9-KO (n=7) vs. WT (n=6), Mann-Whitney U-test.

Supplementary Table 2. The full table of HPLC measurements of the monoamines tissue content.

Cortex (ng/mg tissue)							
**Metabolite**	**WT**			**TAAR9-KO**			**P value**
	**Mean**	**SEM**	**N**	**Mean**	**SEM**	**N**	
**5-HT**	0,054	0,007	11	0,051	0,009	11	0,898
**5-HIAA**	0,016	0,003	11	0,017	0,002	11	0,562
**5-HIAA/5-HT**	0,304	0,046	11	0,479	0,139	11	0,519
**DA**	0,123	0,031	11	0,078	0,037	11	0,152
**DOPAC**	0,048	0,008	11	0,044	0,009	11	0,842
**DOPAC/DA**	0,048	0,008	11	0,044	0,009	11	0,842
**HVA**	0,023	0,004	11	0,026	0,003	11	0,599
**HVA/DA**	0,257	0,058	11	0,505	0,082	11	**0,033***

Striatum (ng/mg tissue)							
**Metabolite**	**WT**			**TAAR9-KO**			**P value**
	**Mean**	**SEM**	**N**	**Mean**	**SEM**	**N**	
**5-HT**	0,15	0,05	11	0,14	0,02	11	0,605
**5-HIAA**	0,09	0,02	11	0,09	0,02	11	0,606
**5-HIAA/5-HT**	0,85	0,12	11	0,64	0,09	11	0,282
**DA**	19,67	3,49	11	21,36	2,01	11	0,426
**DOPAC**	4,65	0,68	11	5,13	0,43	11	0,606
**DOPAC/DA**	0,28	0,05	11	0,25	0,03	11	>0,999
**NE**	1,50	0,35	11	2,24	0,35	11	0,099
**HVA**	1,09	0,13	11	1,22	0,08	11	0,557
**HVA/DA**	0,85	0,12	11	0,64	0,09	11	0,282

**Hippocampus (ng/mg tissue)**							
**Metabolite**	**WT**			**TAAR9-KO**			**P value**
	**Mean**	**SEM**	**N**	**Mean**	**SEM**	**N**	
**5-HT**	0,014	0,002	9	0,027	0,003	10	**0,003****
**5-HIAA**	0,038	0,005	10	0,044	0,008	11	0,605
**5-HIAA/5-HT**	2,859	0,501	9	1,786	0,504	10	**0,035***
**DA**	0,020	0,020	9	0,026	0,007	9	0,340
**DOPAC**	0,020	0,003	9	0,029	0,006	11	0,456
**DOPAC/DA**	1,380	0,339	7	1,164	0,264	11	0,596
**NE**	0,180	0,034	10	0,243	0,051	10	0,529
**HVA**	0,030	0,008	10	0,023	0,005	10	0,631
**HVA/DA**	1,610	0,435	8	0,821	0,191	10	0,101

**Olfactory tubercle (ng/mg tissue)**							
**Metabolite**	**WT**			**TAAR9-KO**			**P value**
	**Mean**	**SEM**	**N**	**Mean**	**SEM**	**N**	
**5-HT**	0,209	0,017	10	0,234	0,042	11	0,918
**5-HIAA**	0,061	0,007	11	0,085	0,023	11	0,519
**5-HIAA/5-HT**	0,339	0,043	11	0,346	0,033	11	>0,999
**DA**	6,328	0,720	11	5,014	0,509	11	0,217
**DOPAC**	1,575	0,209	11	1,406	0,170	11	0,699
**DOPAC/DA**	0,270	0,036	11	0,290	0,028	11	0,519
**NE**	0,718	0,089	11	0,641	0,068	11	0,797
**HVA**	0,395	0,395	11	0,395	0,034	11	0,847
**HVA/DA**	0,066	0,066	11	0,069	0,005	10	0,605

Cortical tissue level of DA and HVA had minimal alterations, with significant changes in dopamine turnover (HVA/DA ratio, $p = 0.033$). Hippocampal levels of 5-HT ($p = 0.003$) and 5-HT turnover (5-HIAA/5-HT ratio, $p = 0.035$) were significantly changed. No significant differences were observed in striatal and olfactory tubercle levels of monoamines.
Data are presented as mean ± SEM. *$p<0.05$, **$p<0.005$ TAAR9-KO vs. WT, Mann-Whitney U-test.

Supplementary Figure 1. Comparative osmotic EFT analysis of WT (n=9) and TAAR9-KO (n=10) rats on 541 nm (A), 555 nm (B), and 577 nm (C) wavelengths.

A.

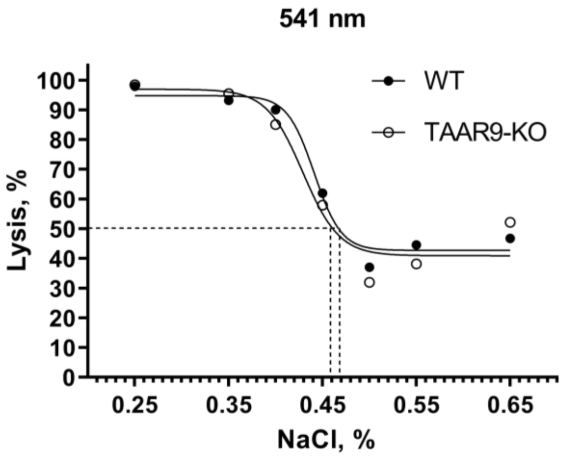

B.

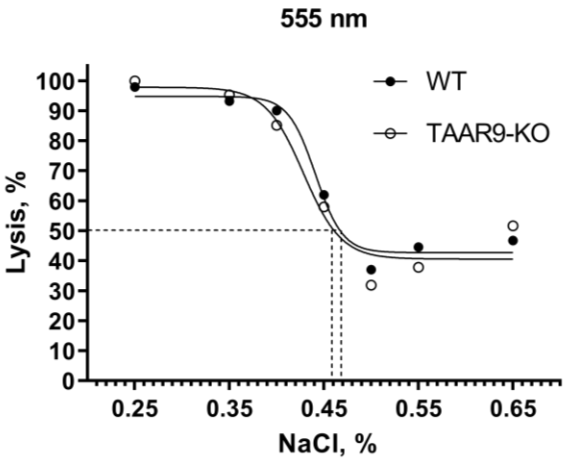

C.

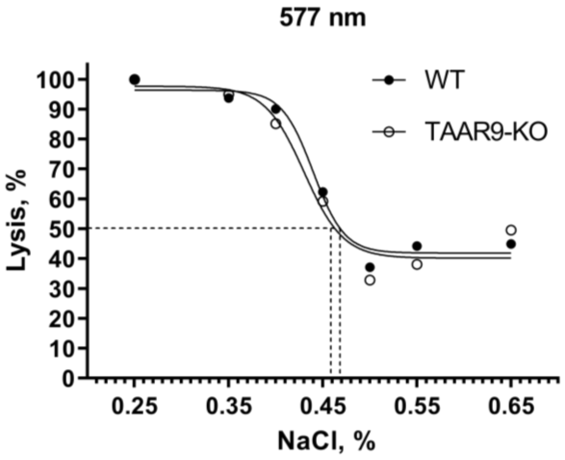

No significant differences were observed at any concentration point between TAAR9-KO and WT rats (50% lysis concentration point: WT - 0.468, KO - 0.459).
